# Breakfast frequency, lifestyle-related factors and their association with body weight status among Polish primary school children aged 10 to 12 years: results from a nationwide cross-sectional study

**DOI:** 10.1186/s12937-025-01231-4

**Published:** 2025-10-21

**Authors:** Krystyna Gutkowska, Elzbieta Wierzbicka, Dawid Madej, Ewa Czarniecka-Skubina, Jadwiga Hamulka

**Affiliations:** 1 Department of Food Market and Consumer Research, Institute of Human Nutrition Sciences, Warsaw University of Life Sciences (WULS-SGGW), Warsaw, Poland; 2 Department of Human Nutrition, Institute of Human Nutrition Sciences, Warsaw University of Life Sciences (WULS-SGGW), Warsaw, Poland; 3 Department of Food Gastronomy and Food Hygiene, Institute of Human Nutrition Sciences, Warsaw University of Life Sciences (WULS-SGGW), Warsaw, Poland

**Keywords:** Breakfast consumption, Frequency, Anthropometric parameters, Lifestyle, Screen time, Sleep duration, Schoolchildren

## Abstract

**Background:**

Concerns about the association between breakfast consumption, lifestyle factors and childhood obesity are increasing. Evidence suggests that regular breakfast intake may play a crucial role in weight management. The present study investigated the association between breakfast frequency, screen time, sleep duration, physical activity, and weight status in schoolchildren.

**Methods:**

A nationwide cross-sectional study was conducted, involving a total sample of 7763 Polish schoolchildren (50.8% girls) aged 10–12 years. Dietary data were collected using the Food Frequency Consumption and Nutritional Knowledge Questionnaire (SF-FFQ4PolishChildren^®^). Trained investigators collected the anthropometric measurements, which were compared to age- and sex-adjusted reference values. Sociodemographic and lifestyle-related data were also collected. Multiple logistic regression analysis was used to examine the association between regular breakfast consumption and weight status, and the mediating effects of lifestyle-related factors confirmed path effects.

**Results:**

Approximately two-thirds of the children were daily breakfast consumers (7 d/wk), 24% were breakfast skippers (0-to-3 d/wk), and 14% had irregular breakfast consumption (4-to-6 d/wk). Younger children were more likely to consume breakfast daily than older children (OR = 0.84, 95%CI: 0.74–0.95; *p* = 0.006). Additionally, children who ate breakfast daily were more physically active than those insufficiently active (OR = 1.16, 95%CI:1.05–1.36; *p* = 0.039) and had lower odds of being overweight or obese compared to those not eating breakfast daily (OR = 0.73, 95%CI: 0.64–0.83; *p* < 0.001). Female children were less likely to be daily breakfast eaters compared to males (OR = 0.74, 95%CI: 0,67–0.82; *p* < 0.001). Children with adequate sleep duration were more likely to eat breakfast daily than those with insufficient sleep (OR = 2.20, 95%CI: 1.85–2.63; *p* < 0.001). Moreover, children with prolonged screen time (> 4 h/day) had lower odds of regular breakfast intake compared to those with screen time of up to 2 h/day (OR = 0.72, 95%CI: 0.63–0.82; *p* < 0.001).

**Conclusions:**

Daily frequency of breakfast consumption was associated with more favourable anthropometric outcomes and lower odds of excessive body weight. Maintaining a healthy lifestyle – incorporating physical activity, optimal sleep duration, limited screen time, and shared meals at school and with family – plays an important role in supporting overall health and weight management in school-aged children. Educational and intervention programmes aimed at preventing or treating obesity in schoolchildren should prioritise regular breakfast consumption alongside other lifestyle-related factors.

**Supplementary Information:**

The online version contains supplementary material available at 10.1186/s12937-025-01231-4.

## Background

Adequate nutrition and physical activity levels are the most important determinants of health during childhood and throughout all the stages of our life cycle [1], and they play a crucial role in preventing being overweight and obesity [2]. The school-age period is also a critical time for acquiring new skills and shaping eating habits that influence health outcomes in later life [3]. In recent years, there has been increasing scientific interest in chrononutrition behaviours, particularly meal timing, frequency, and regularity [4]. Among these dimensions, breakfast frequency has received considerable attention for its potential association with body weight status and metabolic health in children and adolescents [5].

Breakfast is widely regarded as an essential meal of the day, as its regular consumption provides essential energy and nutrients following an overnight fast, thereby contributing to overall well-being [6, 7]. It plays a key role in energy balance, appetite regulation, and reducing the likelihood of excessive energy intake later in the day [8]. However, it remains uncertain whether the relationship between breakfast consumption and body weight is driven by differences in total energy intake or influenced by other lifestyle factors associated with breakfast consumption [9].

For school-aged children, breakfast is particularly important as it supports their growth and physical development [10], mental health [11], improves physical and cognitive function [12], and enhances academic performance, leading to better motivation and achievement [13]. These findings highlight the importance of regular breakfast consumption in promoting optimal nutrition and overall health among children and adolescents. This understanding may serve as a key public health message aimed at fostering greater life satisfaction within these age groups [14]. A well-balanced breakfast is crucial for adequate nutrient intake and is consistently associated with improved food intake and enhanced diet quality in children and adolescents [15, 16]. Notably, regular breakfast eating may contribute to improved postprandial glycaemic response and enhanced insulin sensitivity [17].

Correspondingly, some studies show that breakfast skippers tend to have lower overall energy intake but a higher likelihood of consuming unhealthy snacks later in the day [18], leading to an increased intake of saturated fats, added sugars, and processed foods [7]. In contrast, regular breakfast eaters are more likely to consume nutrient-dense foods contributing positively to their daily energy and nutrient balance [19]. In this context, breakfast frequency has been associated with better overall health, which may lead to better metabolic regulation and, therefore, better long-term weight control and cardiometabolic outcomes [20], although not all studies confirm this association [6, 21].

Current evidence indicates that the prevalence of daily breakfast consumption among children and adolescents varies significantly across different regions. The Health Behaviour in School-aged Children (HBSC) study, conducted across 23 participating countries, reported daily breakfast rates of approximately 80% in Nordic countries, while rates in southern European countries were below 60%. In Poland, daily breakfast consumption stood at 58.8% for boys and 53.2% for girls [22]. The latest HBSC survey (2018–2022), conducted among adolescents (11, 13, and 15-year-olds) across 44 countries and regions in Europe, Central Asia and Canada, revealed that only 51% of adolescents ate breakfast daily, with boys (56%) surpassing girls (46%), and over half of the countries experiencing a decline [23]. Breakfast skipping is more prevalent among adolescents compared to younger children (7–9 years) [24]. Research from the WHO study European Childhood Obesity Surveillance Initiative (COSI) indicates that 78.5% of children eat breakfast daily, with a higher rate (84.5%) reported for Polish children. A higher prevalence of breakfast skippers was reported among adolescents compared to children because breakfast skipping increased during the transition to adulthood. A national study showed that 30% of adolescents (11–13 years) did not eat breakfast daily [25]. Other studies revealed that 40% of older adolescents (13–19-year-olds) skipped breakfast [26], compared with only 22.5% of children 7–10-year-olds [27].

Several factors influence breakfast skipping, including age, gender, frequency of breakfast with family, cultural background, and socioeconomic status. With regard to sex-specific effects, several studies have reported that girls, both children and adolescents, are more likely than boys to skip breakfast. This difference has been partly attributed to factors such as greater concerns about body image, dieting behaviours, and social pressures among girls. However, these findings are not consistent across all studies, with some reporting no significant sex-based differences in breakfast consumption habits [28, 29]. Family habits also play important role; eating breakfast with family was positively associated with adolescents’ breakfast consumption, with more frequent family meals linked to higher diet quality in children [30, 31]. Additionally, low economic status was found to contribute to higher weight, partly due to poor-quality breakfasts that often included energy-dense, high-glycaemic-index foods such as industrially produced baked goods, sugar-sweetened beverages, and fried products [32].

Breakfast consumption is also closely linked to lifestyle factors, including physical activity, sleep, and screen time [33, 34]. Multiple studies suggest that regular breakfast intake plays a crucial role in maintaining a healthy lifestyle, optimal sleep duration, and limited screen time [35], particularly when combined with adequate physical activity [36]. These factors collectively support energy metabolism, cognitive function, and overall well-being in children and adolescents [37, 38]. Physical activity levels also play a crucial role in dietary habits [39]. In particular, studies have demonstrated that regular breakfast consumption is linked to higher levels of physical activity and improved energy balance. Conversely, skipping breakfast is associated with lower energy expenditure and more sedentary behaviour, with contributes to increased body weight [40, 41]. The relevance of breakfast frequency has been highlighted in a recent comprehensive systematic review and meta-analysis which revealed that daily breakfast consumption (7 times/week) was associated with a significantly lower risk of childhood overweight/obesity compared with non-daily breakfast consumption (≤6 times/week) [4]. Similarly, another systematic review found that skipping breakfast in the paediatric population may be an easy indicator assessment of the risk of excess weight gain and unfavourable metabolic outcomes [42]. Aligning daily breakfast consumption, food quality, and quantity with circadian rhythms may optimize metabolic homeostasis [43], and promote overall health for school-aged children [44].

However, despite growing concerns about childhood obesity and diet-related diseases, there is few nationwide research on the precise effect of breakfast frequency on the anthropometric outcomes in Polish school-aged children. This knowledge gap hinders the development of targeted public health interventions and effective nutritional policies, which could potentially exacerbate long-term health risks for this vulnerable population. Therefore, it is important to consider the impact of breakfast eating habits. This study aims to investigate the association between breakfast frequency, lifestyle-related factors and anthropometric parameters, with a focus on regular daily consumption in a nationally representative sample of 10–12-year-old schoolchildren.

## Methods

### Study design, population and recruitment

**Fig. 1:**
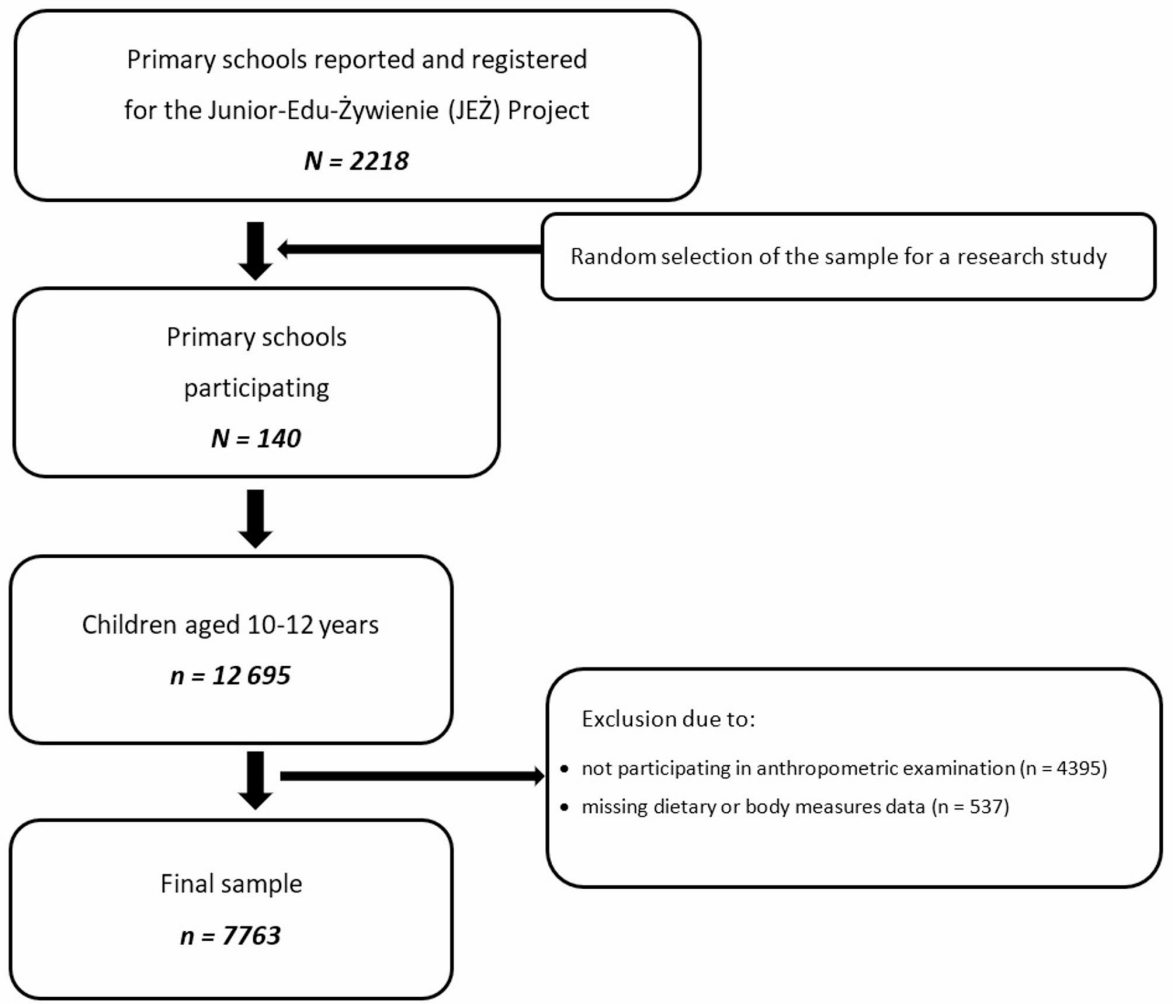
Flow chart of sample selection from the study population. *N* – number of schools, *n* – number of children

The cross-sectional study was conducted in 2022–2023 as part of the National Junior-Edu-Żywienie (JEŻ) Project. Using a two-stage cluster sampling design, 140 schools were selected from 2218 qualified primary schools across Poland, from which selected classes were drawn from each school, numbering a total of 12,695 10–12-year-old schoolchildren. From this sample 7763 children (3820 boys and 3943 girls) were qualified to participate in the study, and questionnaire responses, parental consent, and all necessary anthropometric measurements were obtained from all participants. Detailed information on the study design, methods, and sampling has been described in the protocol study [45]. The recruitment process flowchart is shown in Fig. 1.

### Data collection and measurements

#### Breakfast consumption, eating habits and nutritional knowledge data

The questionnaire consisted of questions regarding socio-demographic factors, e.g., gender, age, place of residence, lifestyle including screen time, sleep duration, physical activity, eating habits, and nutritional knowledge. The questionnaires were collected using a validated tool designed for Polish adolescents: SF-FFQ4Polish-Children^®^ [46]. The paper-and-pencil questionnaire was completed by children in classrooms under the supervision of the researchers and a teacher. Detailed information on this study’s protocol and methodology has been previously published [45].

Respondents were asked about their breakfast-eating practices, e.g. the frequency of eating breakfast during the week and breakfasts eaten at school and on weekends or non-school days. They were also asked about the number of days per week they ate a meal at school and the frequency of eating meals with their families. They could choose from the following answers: not at all; less than 1 time/week; 1–2 days/week; 2–4 days/week; 5–6 days/week; or every day. This questionnaire was described in the study protocol [45]. In the present study, a daily breakfast (DB) was defined as eating breakfast 7 days a week. An irregular breakfast (IR) meant eating 4–6 days a week. Skipping breakfast (SB) was considered as 0–3 days a week. Daily school (DS) meal consumption was defined as eating a regular meal at school (5 days/week), regardless of whether it was home-cooked or served at school.

Children’s eating habits were assessed using a food frequency questionnaire (covering the period of the last 12 months). Respondents could choose from one of the following categories: never or almost never, less than once a week, once a week, 2–4 times/week, 5–6 times/week, every day, several times a day. To comprehensively assess dietary habits in an a priori approach based on the usual frequency of food consumption within last 12 months, three diet quality indicators were calculated: pHDI (pro-Healthy Diet Index): included the consumption of 6 food groups with a potentially beneficial impact on health: dairy products, whole grain products, fish, pulses, vegetables, and fruits; nHDI (non-Healthy Diet Index): included the consumption of 5 food groups with a potentially negative impact on health: fast foods, salty snacks, sweets, sugar-sweetened beverages, and energy drinks; DQI (Diet Quality Index): included 11 food groups (6 with a potentially beneficial and 5 with a potentially negative impact on health). The pHDI and nHDI indices were calculated by adding together the daily consumption frequencies of individual food groups, taking into account the frequency of consumption (frequency/day). The detailed calculation method was presented in the study protocol [45].

Nutrition knowledge was assessed by means of 20 closed questions: 5 questions were taken from the validated SF-FFQ4PolishChildren^®^ questionnaire for Polish adolescents [46], and 15 questions were based on current dietary guidelines for children and adolescents in Poland [47]. Each correct answer was worth 1 point, while the answers ‘I don’t know’, a wrong answer, or no answer were worth 0 points. The score for each participant was calculated as the sum of the scores obtained, ranging from 0 to 20. Based on the tertiles of the distribution of scores, participants were classified into one of three groups of nutrition knowledge: low (0–7 points), medium (8–14 points), and high (15–20 points).

#### Screen and sleep time, and physical activity data

Screen time (ST) was assessed based on the children’s responses to a questionnaire about the time spent in front of computers and electronic devices during the week. Participants selected their average daily screen time from the following options: <2 h/day; 2 to < 4 h/day; 4 to < 6 h/day; 6 to < 8 h/day; 8 to < 10 h/day; and ≥ 10 h/day. The analysis of results included the categories < 2 h/day, 2–4 h/day, and >4 h/day; categories over 4 h were combined due to the small number of responses. The references were determined based on the recommendation of the American Academy of Pediatrics, which suggests that ST for children and adolescents should be a maximum of 2 h/day [48]. Sleep duration was also assessed using a questionnaire in which participants could choose between specific time intervals: < 6 h/day, 6–8 h/day, and >8 h/day. According to the National Sleep Foundation, a sleep deficit is defined as less than 8 h/day for school-aged children [49].

For leisure-time physical activity (after school and at weekends), a self-report measure was used with one of the following three categories: low, moderate, or vigorous. Each response was illustrated with specific examples; low activity: mainly sedentary activities (watching TV, using a computer, reading) or short walks, up to 2 h/week; moderate activity: walking, cycling, gymnastics, light physical activity carried out for 2–3 h/week; vigorous activity: running, cycling, or other sports activities for more than 3 h a week [45].

#### Anthropometric measures and nutritional status

Anthropometric measurements such as body mass (BM), height (H), and waist circumference (WC) were measured using standardized procedures and equipment in accordance with the International Standards for Anthropometric Assessment recommendations [50]. Body Mass Index (BMI, kg/m^2^) and Waist-to-Height Ratio (WHtR) were calculated from the obtained data. Each participant’s BMI value was categorised as follows: underweight, normal body weight, and overweight or obese, according to the national age- and sex-adjusted BMI cut-off criteria for children and adolescents [51], and abdominal obesity measure as a WHtR ≥ 0.5 [52]. BMI z-scores were calculated to categorise children as underweight (BMI z-score <−1.0), normal weight (BMI z-score between − 1.0 and 1.0), and overweight/obese (BMI z-score >1.0) [51]. More details on the study protocol and methods were described previously [45].

### Statistical analysis

The statistical analyses were performed using the SAS 9.4 statistical package (SAS Institute, Cary, NC, USA). The descriptive data were presented as mean values ± standard deviation (SD) or median (Q1; Q3) for continuous variables, and as n (%) for categorical variables according to breakfast frequency categories. The Chi-square test was used to determine statistically significant differences between categorical variables and groups. Based on the Kolmogorov-Smirnov test the hypothesis of normality of continuous variables was rejected; therefore, the Mann-Whitney U test and Kruskal-Wallis test were used to determine statistically significant differences between two and three groups, respectively. The C-statistic value and the Hosmer and Lemeshow Goodness-of-Fit test were used to assess the quality of the resulting model. For all the tests, *p* ≤ 0.05 was considered significant.

To investigate the association between sociodemographic and lifestyle-related factors and daily breakfast (DB) consumption (model 1) and the combined DB at home and daily school (DS) breakfast or lunch consumption (model 2) as dependent variables, multiple logistic regression analysis was performed. Model 1: Regular breakfast intake was defined as consuming breakfast daily (7 days per week). Model 2: To assess the overall regular breakfast consumption, the combined DB and DS (5 days per week) intake was analysed. A logistic regression model with a 95% confidence level (95% CI) was applied to estimate adjusted odds ratios (OR). Wald’s test was used to assess the significance of ORs. Important confounding factors were taken into account, including age, sex, place of living, diet quality indicators (pHDI, nHDI, DQI), eating meals with family, anthropometric parameters, screen time, sleep duration, and physical activity level. After adjustment for the potential confounding variables, further analysis was performed to identify the factors significantly associated with regular/daily breakfast consumption. Associations were considered significant at a *p*-value of ≤0.05.

## Results

### Study participant characteristics

Table 1 presents sociodemographic and anthropometric data. The study included 50.8% girls and 49.2% boys, mostly 10–year-olds (40.2%). Urban residents accounted for 78.7%, with 23.5% living in cities of 100k–500k people. Being overweight or obese was found in 15.3% of the participants, being underweight in 11.5%, and the rest had a normal body weight, while abdominal obesity adiposity was observed in 16.8% of participants. Almost 10% of children slept less than 6 h per day, while 50% had a short sleep duration (6 to < 8 h). More detailed descriptive characteristics of the study participants by gender are presented in Additional Files 1 and 2 (see the Supplementary material).

**Table 1:** Sociodemographic and anthropometric characteristics of the study population by breakfast frequency categories (*n* = 7763)

Variables	Total *n* = 7763	DB *n* = 4863	IB *n* = 1064	SB *n* = 1836	*p*- Value*	DB + DS *n* = 3607	Non-(DB + DS) *n* = 4156	*p*- Value**
Age								
10 years	3117 (40.2)	2056 (42.3)	383 (36.0)	678 (36.9)	< 0.001	1594 (44.2)	1523 (36.7)	< 0.001
11 years	2540 (32.7)	1600 (32.9)	352 (33.1)	588 (32.0)		1160 (32.1)	1380 (33.2)	
12 years	2106 (27.1)	1207 (24.8)	329 (30.9)	570 (31.1)		853 (23.7)	1253 (30.1)	
Gender								
Male	3820 (49.2)	2501 (51.4)	511 (48.0)	808 (44.0)	< 0.001	1791 (49.7)	2029 (48.8)	0.464
Female	3943 (50.8)	2362 (48.6)	553 (52.0)	1028 (56.0)		1816 (50.3)	2127 (51.2)	
Place of living								
Village	1651 (21.3)	972 (20.0)	227 (21.3)	452 (24.6)	< 0.001	722 (20.0)	929 (22.4)	< 0.001
City < 20 k	1393 (17.9)	867 (17.8)	196 (18.4)	330 (18.0)		647 (17.9)	746 (18.0	
City 20–100 k	1530 (19.7)	881 (18.2)	247 (23.3)	402 (21.8)		632 (17.6)	898 (21.5)	
City 100–500 k	1825 (23.5)	1179 (24.2)	232 (21.8)	414 (22.6)		827 (22.9)	998 (24.0)	
City > 500 k	1364 (17.6)	964 (19.8)	162 (15.2)	238 (13.0)		779 (21.6)	585 (14.1)	
Anthropometric data								
Body weight (kg)	42.6 ± 11.8	41.4 ± 11.3 a	43.2 ± 11.4 b	45.3 ± 12.7 c	< 0.001	41.1 ± 11.4	43.8 ± 11.9	< 0.001
Height (cm)	149.9 ± 8.9	149.3 ± 8.9 a	150.7 ± 8.8 b	151.0 ± 9.0 b	< 0.001	149.1 ± 8.9	150.1 ± 8.9	< 0.001
Waist circumference (cm)	66.0 ± 10.4	65.6 ± 10.1 a	66.1 ± 9.7 b	68.0 ± 11.1 c	< 0.001	65.0 ± 10.0	66.9 ± 10.6	< 0.001
BMI (Z-score)	0.008 ± 1.0	−0.08 ± 0.99 a	0.02 ± 1.04 b	0.24 ± 1.09 c	< 0.001	−0.10 ± 0.99	0.10 ± 1.06	< 0.001
WHtR (ratio)	0.44 ± 0.06	0.44 ± 0.06 a	0.44 ± 0.06 a	0.45 ± 0.07 b	< 0.001	0.43 (0.06)	0.45 (0.06)	< 0.001
BMI (category)								
Underweight	888 (11.4)	629 (12.9)	111 (10.4)	148 (8.1)	< 0.001	488 (13.5)	400 (9.6)	< 0.001
Normal weight	5687 (73.3)	3608 (74.2)	793 (74.5)	1286 (70.0)		2673 (74.1)	3014 (72.5)	
Overweight or obesity	1188 (15.3)	626 (12.9)	160 (15.1)	402 (21.9)		446 (12.4)	742 (17.9)	
WHtR (category)								
WHtR < 0.5	6459 (83.2)	4116 (84.6)	902 (84.8)	1441 (78.5)	< 0.001	3086 (85.6)	3373 (81.2)	< 0.001
WHtR ≥ 0.5	1304 (16.8)	747 (15.4)	162 (15.2)	395 (21.5)		521 (14.4)	783 (18.8)	

Continuous variables are presented as mean ± *SD *standard deviation, category variables are presented as count (*n*) and proportion (%). Daily breakfast (DB): 7 days/week, irregular breakfast (IR): 4–6 days/week; skipping breakfast (SB): 0–3 days/week, Daily breakfast (DB) and daily school (DS) meal consumption, (*DB + DS*) Non-daily breakfast (non-DB) and non-daily school (non-DS) consumption: non-(DB + DS), *BMI* Body Mass Index, *WHtR* Waist-to-Height Ratio

*p-values were calculated using the Kruskal-Wallis test for continuous variables and the Chi-squared test for categorical variables

**p-values were calculated using the Mann-Whitney’s test for continuous variables and the Chi-squared test for categorical variables. a, b,c – significant differences between the study groups, p ≤ 0.05 (the Kruskal-Wallis test)

### Breakfast frequency

The study group was divided according to the frequency of breakfast consumption. Among the studied subgroups (Table 1), daily breakfast (DB) was reported by 62%, irregular breakfast (IR), i.e., 4-to-6 days per week, by 14%, and skipping breakfast (SB), i.e. 0-to-3 days per week, by 24% of children. Regular daily consumption of breakfast or lunch at school (DB + DS) was noted in of 46% children, whereas irregular consumption (non-(DB + DS)) was reported by the majority of the study group (54%).

### Characteristics of the study population in relation to breakfast frequency

Table 1 shows sociodemographic and anthropometric data across the subgroups of breakfast frequency. In the group of DB eaters, most frequently identified were children 10–year-olds (42.3%), boys (51.4%) and children living in large cities with a population of 100–500 thousand (23.5%). Irregular breakfast (IB) was also reported mostly by children aged 10 but also by girls (52%) and children living in medium-sized cities with 20–100 thousand inhabitants (23.3%). Skipping breakfast (SB) was most frequently observed in the group of 10–year-old children (36.9%), girls (56.0%), and children living in rural areas (24.6%).

Statistically significantly higher mean body mass, height, waist circumference and BMI values were observed in the SB group compared to the DB and IB groups. These values were significantly higher in the non-(DB + DS) group than in the DB + DS group. The SB group also had a significantly higher WHtR ratio and the highest percentage of children with a WHtR greater than or equal to 0.5 (indicating abdominal obesity adiposity) compared with the other subgroups (Table 1).

**Fig. 2:**
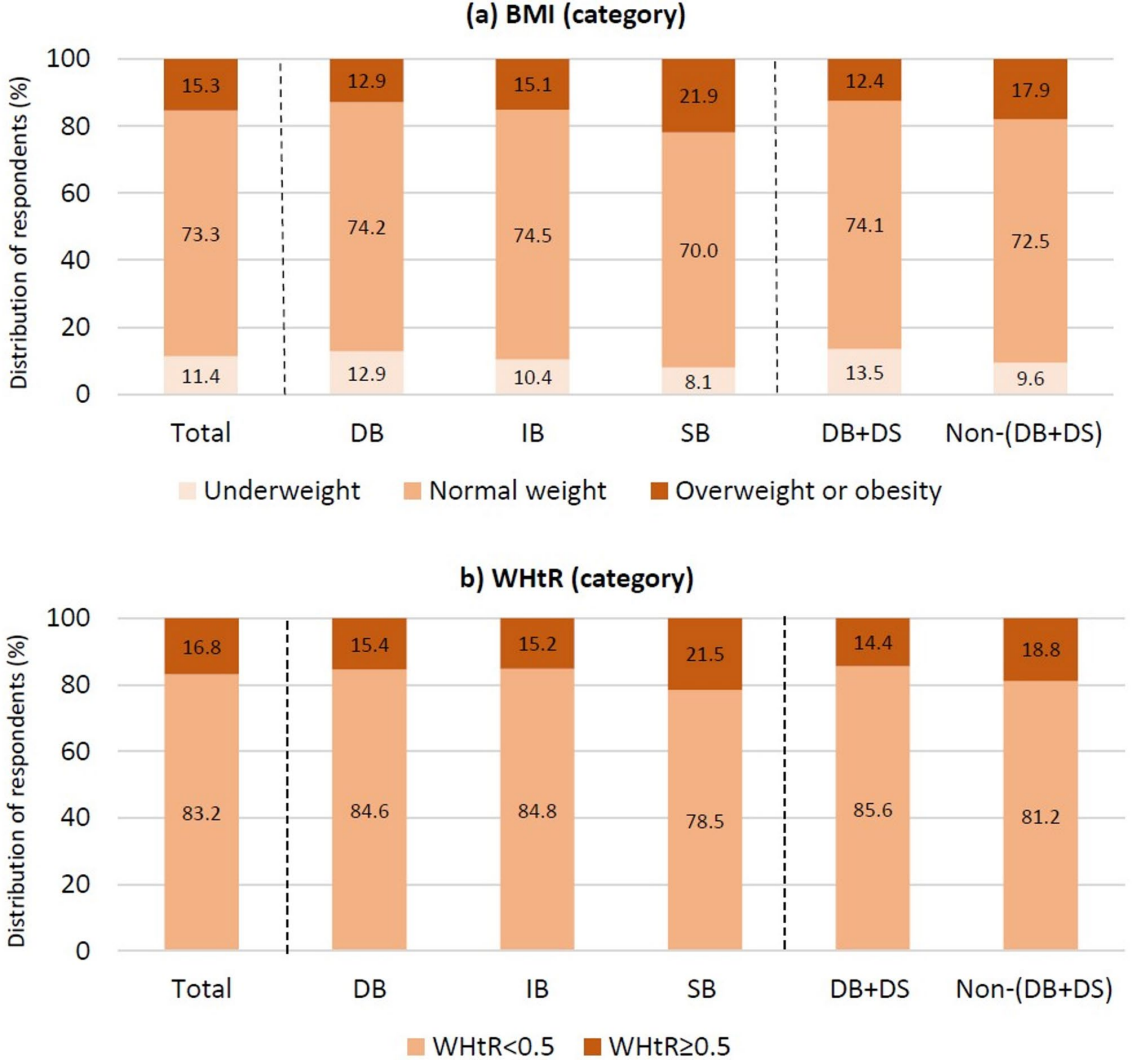
Distribution of respondents by breakfast frequency categories in relation to selected anthropometric data: **a** BMI; **b** WHtR. Notes: Daily breakfast (DB): 7 days/week; irregular breakfast (IR): 4–6 days/week; skipping breakfast (SB): 0–3 days/week; Daily breakfast (DB) and daily school (DS) meal consumption: (DB + DS); Non-daily breakfast (non-DB) and non-daily school (non-DS) consumption: non-(DB + DS)

The highest prevalence of children classified as overweight or obese was observed in the SB group (21.9%) while the lowest was in the DB group (12.9%). Similarly, a higher percentage of overweight and obese children was found in the non-(DB + DS) group (17.9%) compared to the DB + DS group (12.4%) (Fig. 2).

The SB group also had a significantly higher WHtR ratio and the highest percentage of children (21.5%) with a WHtR ≥ 0.5, indicating abdominal adiposity. In contrast, the prevalence of abdominal adiposity was 15.4% in the DB group and 15.2% in the IB group (Table 1; Fig. 2). Furthermore, a higher percentage of children with abdominal adiposity was found in the non-(DB + DS) group (18.8%) compared to 14.4% in the DB + DS group (Fig. 2).

### Dietary quality and nutritional knowledge in relation to breakfast frequency

Statistically significantly lower DQI (Diet Quality Index) and pHDI (pro-Healthy Diet Index) were observed in the SB group compared to the DB and IB groups (Table 2). At the same time, the nHDI (non-Healthy Diet Index) was significantly higher in the SB group than in the other groups. The non-(DB + DS) group also had significantly lower DQI and pHDI and higher nHDI than the DB + DS group. However, about 65.1% of the children reported eating a meal at school every day, while 7.4% of the participants ate a meal at school less than once a week. Only 40% of the children reported eating meals with their families every day. In particular, those with daily breakfast consumption displayed a significantly higher diet quality than the skippers (*p* < 0.001). The majority of children in all groups had a moderate level of nutrition knowledge (Table 2).

**Table 2:** Diet quality indicators, selected eating habits and knowledge of nutrition in the study population according to frequency of breakfast consumption (*n* = 7763)

Variables	Total *n* = 7763	DB *n* = 4863	IB *n* = 1064	SB *n* = 1836	*p*- value*	DB + DS *n* = 3607	Non-(DB + DS) *n* = 4156	*p*- value**
Diet quality indices pHDI (score)								
pHDI (score)	18.8 (13.5–26.7)	20.8 (14.2–28.5) a	18.2 (12.7–26.0) b	17.7 (11.2–25.0) c	< 0.001	21.3 (14.7–29.7)	17.8 (11.7–25.5)	< 0.001
nHDI (score)	9.2 (5.6–16.2)	8.4 (5.0–15.6.0.6) a	11.2 (6.2–16.4) b	11.2 (5.6–16.4) b	< 0.001	8.4 (5.0–15.6.0.6)	10.6 (5.6–16.4)	< 0.001
DQI (score)	8.7 (1.0–17.3.0.3)	10.2 (2.2–18.8) a	7.1 (−0.3-15.4) b	6.1 (−1.6-14.1) c	< 0.001	10.9 (2.8–19.5)	6.8 (−0.7-15.0)	< 0.001
Eating meals at school								
< 1 days/week	571 (7.4)	269 (5.5)	77 (7.2)	225 (12.3)	< 0.001	-	-	-
1–3 days/week	773 (10.0)	267 (5.5)	141 (13.3)	365 (19.9)		-	-	
4–6 days/week	1365 (17.5)	720 (14.8)	299 (28.1)	346 (18.8)		-	-	
Every day	5054 (65.1)	3607 (74.2)	547 (51.4)	900 (49.0)		-	-	
Eating meals with family								
Not at all	253 (3.3)	122 (2.5)	26 (2.4)	105 (5.7)	< 0.001	77 (2,1)	176 (4.2)	< 0.001
< 1 day/week	446 (5.7)	219 (4.5)	48 (4.5)	179 (9.8)		135 (3.7)	311 (7.5)	
1–2 days/week	1151 (14.8)	611 (12.6)	177 (16.6)	363 (19.8)		438 (12.1)	713 (17.2)	
3–4 days/week	1637 (21.1)	912 (18.8)	269 (25.3)	456 (24.7)		611 (16.9)	1026 (24.7)	
5–6 days/week	1254 (16.2)	780 (16.0)	223 (21.0)	251 (13.7)		558 (15.6)	696 (16.7)	
Every day Nutrition knowledge	3022 (38.9)	2219 (45.6)	321 (30.2)	482 (26.3)		1788 (49.6)	1234 (29.7)	
Nutrition knowledge								
Low	2068 (26.6)	1251 (25.7)	256 (24.1)	561 (30.6)	< 0.001	926 (25.7)	1142 (27.5)	< 0.001
Moderate	4676 (60.3)	2894 (59.5)	667 (62.7)	1115 (60.7)		2123 (58.9)	2553 (61.4)	
High	1019 (13.1)	718 (14.8)	141 (13.2)	160 (8.7)		558 (15.5)	461 (11.1)	

Continuous variables are presented as median (Q1–Q3); category variables are presented as count (*n*) and proportion (%). Daily breakfast (DB): 7 days/week, irregular breakfast (IR), 4–6 days/week, skipping breakfast (SB), 0–3 days/week, Daily breakfast (DB) and daily school (DS) meal consumption: (DB + DS), *non-DB* Non-daily breakfast and *non-DS* non-daily school consumption: non-(DB + DS), *pHDI *pro-Healthy Diet Index, *nHDI *non-Healthy Diet Index, *DQI *Diet Quality Index

*p-values were calculated using the Kruskal-Wallis test for continuous variables and the Chi-squared test for categorical variables

**p-values were calculated using the Mann-Whitney’s test for continuous variables and the Chi-squared test for categorical variables. a, b,c – significant differences between the study groups, *p* ≤ 0.05 (the Kruskal-Wallis test)

### Screen and sleep time and physical activity data in relation to breakfast frequency

**Fig. 3:**
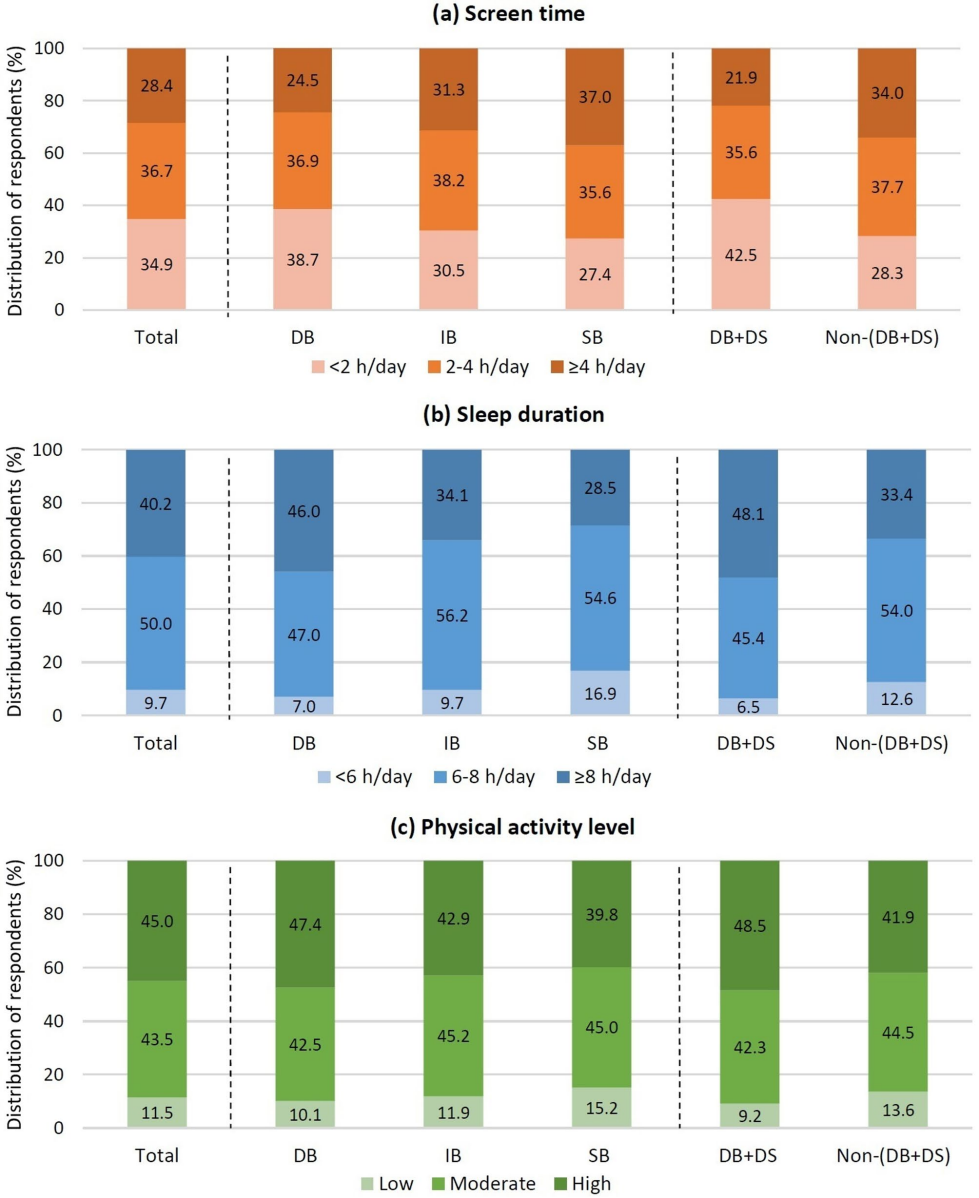
Distribution of respondents by breakfast frequency categories in relation to selected lifestyle-related factors: **a** Screen time; **b** Sleep duration; **c** Physical activity level. Notes: Daily breakfast (DB): 7 days/week; irregular breakfast (IR): 4–6 days/week; skipping breakfast (SB): 0–3 days/week; Daily breakfast (DB) and daily school (DS) meal consumption: (DB + DS); Non-daily breakfast (non-DB) and non-daily school (non-DS) consumption: non-(DB + DS)

A total of 36.7% of children spent between 2 and 4 h per day on screen-based activities (Fig. 3). The highest proportion of those with the longest screen time (≥ 4 h/day) was observed in the SB group (37.0%), while the lowest was recorded in the DB group (24.5%). Meanwhile, the DB + DS group had the highest percentage of children (42.5%) with the shortest screen time.

Most children slept 6–8 h per day (50%). The highest percentage of children who slept < 6 h/day was observed in the SB group (16.9%), compared to only 7% in the DB group. Similarly, a higher percentage of children sleeping < 6 h was observed in the non-(DB + DS) group (12.6%) compared to 6.5% in the DB + DS group. A high level of physical activity was observed in 45% of participants. As the frequency of breakfast consumption decreased, the proportion of children with high physical activity decreased, and the proportion with low physical activity increased. Regarding the lifestyle parameters, children with regular breakfast consumption were more likely to engage in higher levels of physical activity, spend a shorter period of time looking at a screen, and have a higher sleep duration, as compared to their counterparts, especially in the SB group (Fig. 3).

### Predictive model for daily breakfast consumption (model 1)

Table 3 shows odds ratios (OR), which represent the probability of belonging to a selected breakfast consumption group, i.e. daily eating of breakfast at home (7 days/wk.). Girls were 26% less likely to consume breakfast regularly compared to boys (reference group) (OR: 0.74; 95% CI: 0.67–0.82). The likelihood of regular breakfast consumption decreased with age. Eleven-year-olds were 4% less likely (not statistically significant), and 12-year-olds were 16% less likely to eat breakfast regularly compared to the youngest group (10-year-olds) (OR: 0.84; 95% CI: 0.74–0.95).

**Table 3:** Predictive model for daily/regular breakfast consumption in relation to selected sociodemographic, eating habits and lifestyle-related determinants; model 1 (*n* = 7763)

Variables/Factors	Level	β	OR	95%CI		*p*-value
Intercept		0.202				0.160
Gender	male (ref.)	0	1			
	female	−0.296	0.74	0.67	0.82	< 0.001
Age (years)	10 (ref.)	0	1			
	11	−0.042	0.96	0.85	1.08	0.485
	12	−0.176	0.84	0.74	0.95	0.006
Screen time (average per day)	< 2 h/day (ref.)	0	1			
	2–4 h/day	−0.153	0.86	0.76	0.97	0.013
	> 4 h/day	−0.335	0.72	0.63	0.82	< 0.001
Sleep duration (average per day)	< 6 h/day (ref.)	0	1			
	6–8 h/day.	0.398	1.49	1.26	1.76	< 0.001
	> 8 h/day	0.790	2.20	1.85	2.63	< 0.001
Physical activity level	low (ref.)	0	1			
	moderate	0.111	1.12	1.02	1.32	0.043
	high	0.144	1.16	1.05	1.36	0.039
BMI (category)	normal weight (ref.)	0	1			
	underweight	0.268	1.31	1.11	1.54	0.002
	overweight/obesity	−0.319	0.73	0.64	0.83	< 0.001
Frequency of eating meals at school (breakfast or lunch)	< 1/week (ref.)	0	1			
	every day at school	0,0.781	2.18	1.82	2.63	< 0.001
	3–4/week	0.120	1.13	0.92	1.38	0.253
	1–2/week	−0.581	0.56	0.44	0.70	< 0.001
Frequency of eating meals with family	every day (ref.)	0	1			
	5–6 days/week	−0.426	0.65	0.56	0.76	< 0.001
	3–4 days/week	−0.587	0.56	0.49	0.64	< 0.001
	1–2 days/week	−0.650	0.52	0.45	0.61	< 0.001
	1 day/week	−0.687	0.50	0.41	0.62	< 0.001
	< 1 day/week	−0.637	0.53	0.4	0.70	< 0.001

*OR* point estimate (eβ), 95% *CI* confidence intervals; significance level of the Wald’s test

Screen time was negatively associated with regular breakfast consumption. Children who had 2–4 h of screen time daily were 14% less likely to consume breakfast regularly compared to those with ≤ 2 h (OR: 0.86; 95% CI: 0.76–0.97). For children with over 4 h of daily screen time, the likelihood decreased by 28% compared to the reference group (OR: 0.72; 95% CI: 0.63–0.82). Sleep duration was positively associated with breakfast consumption. Children sleeping 6–8 h per day were 49% more likely to consume breakfast regularly compared to those sleeping less than 6 h (OR: 1.49; 95% CI: 1.26–1.76). For children sleeping 8 or more hours, the likelihood increased by 120% compared to the shortest sleepers (OR: 2.20; 95% CI: 1.85–2.63).

Physical activity was positively associated with regular breakfast consumption. An increase in physical activity from low to moderate raised the likelihood of regular breakfast consumption by 12% (OR: 1.12; 95% CI: 1.02–1.32), while a shift from low to high activity increased the likelihood by 16% (OR: 1.16; 95% CI: 1.05–1.36). Overweight children were 27% less likely to consume breakfast regularly compared to children with ‘normal weight’ (OR: 0.73; 95% CI: 0.64–0.83). In contrast, children who were underweight were 31% more likely to eat breakfast regularly compared to those with ‘normal weight’ (OR: 1.31; 95% CI: 1.11–1.54).

Frequent school meal consumption (packed second breakfast or lunch) appeared to increase the likelihood of regular breakfast intake. However, children consuming a school meal 1–3 times per week were 44% less likely to eat breakfast regularly compared to those consuming school meals less than once per week (reference group) (OR: 0.56; 95% CI: 0.44–0.70). Those consuming school meals 4–6 times per week showed a 13% higher likelihood (not statistically significant), while children eating school meals daily were 118% more likely to eat breakfast regularly compared to the reference group (OR: 2.18; 95% CI: 1.82–2.63).

The less frequently children ate meals with their families, the lower their likelihood of regular breakfast consumption. Children who never ate with their family were 47% less likely to eat breakfast regularly compared to those who ate with their family daily (reference group) (OR: 0.53; 95% CI: 0.40–0.70). For children eating with their family once a week, the likelihood was halved compared to the reference group (OR: 0.50; 95% CI: 0.41–0.62). For those eating together 1–2 times per week, the likelihood decreased by 48% (OR: 0.52; 95% CI: 0.45–0.61), 3–4 times per week by 44% (OR: 0.56; 95% CI: 0.49–0.64), and 5–6 times per week by 35% (OR: 0.65; 95% CI: 0.56–0.76) compared to children eating with their families daily.

OR - point estimate (eβ), 95% confidence intervals (CI); significance level of the Wald’s test.

### Predictive model for combined daily breakfast and school meal consumption (model 2)

Table 4 shows odds ratios (OR) representing the probability of belonging to a selected breakfast consumption group, i.e., daily eating breakfast at home and daily eating meal at school (Model 2). Gender did not show a statistically significant effect on the dependent variable. The likelihood of regular breakfast and school meal consumption decreased with age. For 11-year-olds, the likelihood was 12% lower (OR: 0.88; 95% CI: 0.79–0.98), and for 12-year-olds, it was 22% lower compared to the youngest group (10-year-olds) (OR: 0.78; 95% CI: 0.69–0.88).

**Table 4:** Predictive model for daily/regular meals at school (breakfast or lunch) consumption by selected sociodemographic, eating habits, and lifestyle-related determinants; model 2 (*n* = 7763)

Variables/Factors	Level	β	OR	95%CI		*p*-value
Intercept		−0.016				0.897
Gender	male (ref.)	0	1			
	female	−0.046	0,96	0,87	1,05	0.339
Age (years)	10 (ref.)	0	1			
	11	−0.128	0.88	0.79	0.98	0.023
	12	−0.250	0.78	0.69	0.88	< 0.001
Screen time (average per day)	< 2 h/day (ref.)	0	1			
	2–4 h/day	−0.355	0.70	0.63	0.78	< 0.001
	> 4 h/day	−0.591	0.55	0.49	0.63	< 0.001
Sleep duration (average per day)	< 6 h (ref.)	0	1			
	6–8 h/day	0.461	1.59	1.33	1.89	< 0.001
	> 8 h/day	0.852	2.34	1.96	2.80	< 0.001
Physical activity level	low (ref.)	0	1			
	moderate	0.170	1.19	1.01	1.39	0.038
	high	0.217	1.24	1.06	1.46	0.008
BMI (category)	normal weight (ref.)	0	1			
	underweight	0.269	1.31	1.13	1.52	0.001
	overweight/obesity	−0.281	0.76	0.66	0.86	< 0.001
Frequency of eating meals with family	every day (ref.)	0	1			
	5–6 days/week	−0.546	0.58	0.51	0.66	< 0.001
	3–4 days/week	−0.767	0.46	0.41	0.53	< 0.001
	1–2 days/week	−0.715	0.49	0.42	0.57	< 0.001
	< 1 day/week	−0.904	0.41	0.30	0.54	< 0.001
	1 day/week	−0.964	0.38	0.31	0.48	< 0.001

*OR* point estimate (eβ), 95% *CI* confidence intervals; significance level of the Wald’s test

Children with 2–4 h of daily screen time were 30% less likely to consume breakfast and school meals regularly compared to those with ≤ 2 h (OR: 0.70; 95% CI: 0.63–0.78). For those with more than 4 h of screen time per day, the likelihood decreased by 45% compared to the reference level (OR: 0.55; 95% CI: 0.49–0.63). Sleep duration was positively associated with breakfast and school meal consumption. Children sleeping 6–8 h per day were 59% more likely to consume these meals regularly compared to those sleeping less than 6 h (OR: 1.59; 95% CI: 1.33–1.89). For those sleeping 8 or more hours, the likelihood increased by 134% compared to the shortest sleepers (OR: 2.34; 95% CI: 1.96–2.80).Children who were overweight were 24% less likely to consume breakfast and school meals regularly compared to children with ‘normal weight’ (OR: 0.76; 95% CI: 0.66–0.86).

Physical activity was positively associated with the dependent variable. Increasing activity from low to moderate raised the likelihood of regular breakfast and school meal consumption by 19% (OR: 1.19; 95% CI: 1.01–1.39), while moving from low to high activity increased the likelihood by 24% (OR: 1.24; 95% CI: 1.06–1.46). Screen time had a negative effect on the dependent variable. Conversely, children who were underweight were 31% more likely to consume these meals regularly compared to the reference group (normal weight) (OR: 1.31; 95% CI: 1.13–1.52). The less frequently meals were eaten with family, the lower the likelihood of regular breakfast and school meal consumption.

OR - point estimate (eβ), 95% confidence intervals (CI); significance level of the Wald’s test.

Children who never ate meals with their family were 59% less likely to consume these meals regularly compared to those who ate with their family daily (reference group) (OR: 0.41; 95% CI: 0.30–0.54). For children eating with family once a week, the likelihood was 62% lower compared with the reference group (OR: 0.38; 95% CI: 0.31–0.48). For those eating together 1–2 times per week, the likelihood decreased by 51% (OR: 0.49; 95% CI: 0.42–0.57), 3–4 times per week by 54% (OR: 0.46; 95% CI: 0.41–0.53), and 5–6 times per week by 42% (OR: 0.58; 95% CI: 0.51–0.66) compared to children eating with their family daily.

## Discussion

The main finding of the present nationwide cross-sectional study is that DB consumption is associated with more favourable anthropometric outcomes and healthier lifestyle behaviours, while breakfast skipping is linked to an increased risk of being overweight or obese in 10–12-year-old schoolchildren. Few national studies have examined daily breakfast consumption with weight status [25, 26], therefore, it was key for this study to highlight the importance of lifestyle-related behaviours, and their impact on body weight in a representative sample of Polish schoolchildren from both urban and rural areas. Given the ongoing social changes in the country, collecting updated data is essential to under-standing these relationships.

This study identified significant associations between breakfast frequency and body weight status – overweight or obesity was most prevalent among breakfast skippers, compared to those with irregular or daily breakfast habits. These findings are consistent with a substantial body of existing research that highlights the negative impact of irregular breakfast consumption on anthropometric outcomes [53–56]. Furthermore, our observations on the prevalence of skipping breakfast among schoolchildren are consistent with international and national data, showing a considerable proportion of children and adolescents across various age groups do not consume breakfast daily [24–27]. This emphasises the widespread nature of irregular breakfast habits and their potential public health implications.

Gender differences in breakfast consumption frequency were also observed in our study. Girls were less likely than boys to consume breakfast daily, a finding consistent with previous studies indicating that girls are more likely to skip breakfast due to concerns about body weight, reduced morning appetite, or lifestyle preferences [55, 57, 58]. Given the association between breakfast skipping, poorer nutritional status, and an increased risk of obesity, targeted interventions may be necessary to encourage regular breakfast consumption among girls. Female gender and age over 12 years were identified as significant predictors of skipping breakfast and/or school meals [25]. These findings, consistent with our results, highlight the need for targeted nutritional interventions focusing on nutrition education in these groups. Additionally, studies indicate breakfast skipping is associated with poorer diet quality, irregular meal patterns, and increased consumption of unhealthy foods later in the day [59, 60]. Moreover, they show that inadequate sleep and excessive screen time can disrupt circadian rhythms, leading to metabolic dysregulation [61].

The association between skipping breakfast and a higher risk of overweight and obesity in school-aged children, confirmed in this study, may be due to poor dietary habits, such as unhealthy snacking and increased energy intake later in the day [62–64], as well as lower levels of physical activity [40, 65]. In another study, a positive correlation was found between breakfast consumption and higher physical activity levels [66]. Regular breakfast consumption may, therefore, serve as an indicator of overall healthy eating behaviours, suggesting that lifestyle factors are intricately linked to breakfast habits. They also found that breakfast frequency was correlated with well-being, age, and general health, and a significant correlation with BMI was identified. Our study also revealed that the children who skipped breakfast had a higher waist-to-height ratio, indicating a higher risk of abdominal obesity. These findings are consistent with previous research showing that skipping breakfast is associated with an increased risk of obesity in children and adolescents as well as metabolic syndrome in adulthood [67]. Recent systematic reviews and meta-analyses indicate that skipping breakfast significantly increases the risk of overweight and obesity in children and adolescents, with girls being more vulnerable to this effect [68]. Similar findings from a cross-sectional and cohort study confirm a significant association between low breakfast consumption frequency and increased risk of overweight and obesity. Notably, this effect was not significantly associated with age, sex, geographic region, or economic status, indicating that breakfast skipping is a widespread risk factor for excessive weight gain [69].

In line with our findings, previous intervention studies and randomised controlled trials have shown that skipping breakfast is associated with poorer dietary quality in children and adolescents. Notably, interventions that promote regular breakfast consumption have been effective in reducing its omission. This suggests that breakfast consumption could be a modifiable dietary habit for improving nutritional status and reducing cardiometabolic risk in young respondents [70]. Breakfast skipping may be as an early marker for metabolic dysfunction, especially in those who are overweight, as demonstrated in another study [71]. Diet quality analysis using the DQI indicators in our study demonstrated that breakfast skippers had significantly lower scores in both the overall DQI and the pro-Healthy Diet Index (pHD) compared to daily breakfast consumers. Conversely, the non-Healthy Diet Index (nHDI) was significantly higher among those who skipped breakfast. These results indicate that regular breakfast consumption is associated with healthier dietary patterns, including greater intake of nutrient-dense foods [72] such as whole grains, dairy products, and fruit and vegetables, which are commonly consumed at breakfast [73–75]. In contrast, breakfast skippers may compensate for missed nutrients by consuming nutrient-poor foods later in the day, thereby lowering overall diet quality and increasing the risk of obesity [65]. Children and adolescents who regularly consume breakfast demonstrate higher overall diet quality and better adherence to dietary recommendations compared to those who skip breakfast [25, 56]. These findings suggest that regular breakfast consumption has a beneficial effect on the overall diet quality of children, helps to prevent unhealthy eating behaviours, and may contribute to better weight management.

In our study, in addition to breakfast frequency, eating meals at school was identified as a key factor in maintaining optimal body weight in children. The risk of being overweight and obese was higher among children who did not eat school meals compared to those who did. School meals often provide nutritionally balanced options, ensuring adequate nutrient intake and reducing reliance on unhealthy snacks or processed foods [76]. Similarly, another study has shown that school meal participation is associated with better adherence to dietary recommendations [77]. Moreover, results from other studies suggest that skipping both of breakfast at home and meals at school a few times a week was associated with general adiposity [25]. It should be emphasised that schools are in a unique position to address the promotion and prevention of childhood weight problems and obesity, reaching nearly all children and, to some extent, their families [78].

Our findings also highlight the importance of family meals in shaping children’s dietary behaviours and habits. This is supported by studies indicating that children who eat with their families tend to consume healthier meals, including more vegetables, whole grains, fruit and pulses, as well as fewer sugary drinks and fast food. Family meals also provide structure and social interaction, both of which have been linked to better dietary quality, healthier eating habits and improved weight management [30, 79]. Moreover, regular family meals and breakfast consumption support healthier dietary habits and weight management. They have also been linked to better academic performance in both boys and girls, likely because of their positive impact on health, cognitive function and daily routine [80].

This study also demonstrated the relationship between lifestyle behaviours – including physical activity, sleep duration, and screen time – and dietary habits, particularly breakfast consumption. Children who consumed a more well-balanced diet were more likely to engage in sufficient physical activity, which was associated with higher energy expenditure [39]. Another study demonstrated the beneficial effects of regular breakfast consumption, as it may contribute to a higher intake of fruits and vegetables and is associated with reduced screen time [75]. In contrast, skipping breakfast, often compensated by high-energy intake at dinner and evening snacks, may disrupt metabolic homeostasis and contribute to weight gain, especially in children with low levels of physical activity, as it is associated with an increased body mass index [81].

Moreover, our study shows that children with adequate sleep duration were more likely to eat breakfast daily, whereas those with insufficient sleep had a higher likelihood of skipping breakfast. This finding highlights the critical role that sleep plays in establishing healthy routines and behaviours in children, with adequate rest potentially contributing to better adherence to regular eating patterns. Inadequate sleep has been linked to several negative outcomes, including an increased risk of unhealthy eating behaviours, as children may feel more fatigued, less motivated [82], or more likely to skip meals, including breakfast [33]. The association between insufficient sleep and skipping breakfast with overweight/obesity in children and adolescents has also been confirmed in another study [83].

In our study, prolonged screen time and sleep duration were negatively associated with regular breakfast consumption, further supporting the link between excessive screen use, poor dietary habits, and a sedentary lifestyle. This is consistent with previous research showing how excessive screen time, particularly before bed, can disrupt sleep patterns and reduce the likelihood of engaging in healthy behaviours such as eating regular meals [33, 34, 61, 63]. As screen time increases, children often replace healthy routines, like having a nutritious breakfast with sedentary behaviours like snacking on unhealthy foods while using electronic devices. This sedentary lifestyle, combined with poor dietary habits, can contribute to an increased risk of weight gain and other health problems [84–86]. Previous national studies have also reported that frequent skipping of breakfast and school meals is associated with being overweight and abdominal obesity, particularly among adolescents with higher screen time, and lower physical activity [25], highlighting the need for targeted interventions.

Based on our study, it is worth noting that shifting dietary patterns, environmental factors such as parental eating habits, family meal frequency, easy access to highly processed foods, low physical activity levels (sedentary lifestyle), and insufficient sleep are associated with the prevalence of being overweight and obesity in children. Consequently, they should be considered when developing and implementing intervention and education programmes [87].

The results of our study highlight the importance of promoting daily breakfast consumption as part of a comprehensive strategy to improve overall diet quality in school-aged children and adolescents. Any strategies aimed at maintaining optimal body weight and related interventions should emphasise the role of breakfast in supporting energy balance, enhancing cognitive function, and reducing obesity-related health risks. Additionally, promoting physical activity, adequate sleep duration, reduced screen time, and the regular consumption of meals, both at school and with family, is essential in fostering healthy eating habits.

### Strengths and limitations

The main strength of our study lies in its large sample size, which covers the entire territory of Poland. This large dataset allows for powerful statistical analyses and facilitates the identification of specific associations between variables in adolescents within a age range (10–12 years). In addition, the diverse composition of the sample reflects the demographic and social spectrum of 10–12-year-olds adolescents, supporting generalisations to the wider population. The study was carried out by a qualified team experienced in this type of research. The returned questionnaires were continuously checked and any doubts or shortcomings were clarified with children on an ongoing basis. Furthermore, the statistical models took into account a wide range of factors, including socio-demographic variables (gender, age and place of residence), lifestyle-related factors (leisure-time physical activity, screen time and sleep duration) and eating behaviours. Given the limited national research on daily breakfast consumption and weight status, our study was crucial in highlighting the importance of lifestyle behaviours and their impact on body weight within a representative sample of Polish schoolchildren from both urban and rural areas. Collecting this updated data is essential for understanding these relationships, especially in light of ongoing social changes across the country.

However, the study also has its limitations. First, the cross-sectional study makes it difficult to establish cause and effect relationships, as this type of study provides only a single measure of both the putative cause and effect. Despite this limitation, cross-sectional studies are valuable for collecting data from large populations and comparing differences between groups. They are widely used in research and have inherent advantages and disadvantages that should be carefully considered when designing studies and interpreting results [88]. We also acknowledge the possibility of residual confounding, where unmeasured or imperfectly measured factors could still influence the observed associations, despite our comprehensive efforts to control for a wide range of variables. To assess the dietary habits of adolescents, we used diet quality indicator scores and a short form of the Food Frequency Questionnaire (FFQ), a commonly used tool for this population that has also been validated for children in Poland [46]. Although brief dietary assessment tools have several advantages, such as ease of use and cost-effectiveness, they also have notable limitations. For example, they rely heavily on participants’ recall and are susceptible to social bias, which may have influenced the accuracy of participants’ responses. In addition, the food list in a short FFQ may not capture all items consumed, potentially leading to under-reporting [89, 90]. There was also no data collected on the composition of breakfasts or their nutritional value, which limits our understanding of the quality of the breakfasts consumed.

Moreover, standard proxy measures of adiposity (BMI, WHtR) were used, which are widely used in many epidemiological studies and have well-established procedures for data collection and interpretation in an international context. This can be considered both an advantage, due to their ease of use and comparability across studies, and a limitation, as these measures may not fully capture the complexities of obesity, e.g., body composition assessment. Therefore, in future studies, the use of more advanced methods for measuring obesity should be considered.

## Conclusions

We revealed a higher daily frequency of breakfast consumption was associated with more favourable anthropometric outcomes and lower odds of excessive body weight among schoolchildren. The predictive models developed indicate a significant association between the likelihood of regular breakfast consumption and age (with the probability decreasing with age and parental control diminishes), sex (lower likelihood among girls than boys), sleep duration (increasing with 6–8 h of sleep), physical activity (increasing with activity levels), and the frequency of eating second breakfasts and lunches at school, as well as having meals together with the family (increasing with the frequency of these behaviours). Maintaining a healthy lifestyle – incorporating regular physical activity, optimal sleep duration, limited screen time, and shared meals both at school and within the family environment – plays an important role in supporting the overall health and weight management of school-aged children, particularly girls. Educational and intervention programmes aimed at preventing or managing obesity in this population should prioritise regular breakfast consumption alongside other lifestyle-related factors.

## Supplementary Information

Addtional file. 1: Table 1 Baseline characteristics of the study population (N=7763). Table 2 Dietary outcomes of the study population (N=7763).

## Data Availability

The datasets used and analysed during the current study are available from the first author on reasonable request.
