# Supplementary material for: Breakfast frequency, lifestyle-related factors and their association with body weight status among Polish primary school children aged 10 to 12 years: results from a nationwide cross-sectional study
Source: Nutr J. 2025 Oct 21;24:160. doi: 10.1186/s12937-025-01231-4 (PMC12542513; doi:10.1186/s12937-025-01231-4)
Supplement: Supplementary file 1 — Addtional file. 1: Table 1 Baseline characteristics of the study population (N=7763). Table 2 Dietary outcomes of the study population (N=7763). [file 12937_2025_1231_MOESM1_ESM.docx]

**Additional File 1. Baseline characteristics of the study population (n=7763).**

| Variables | Total  n=7763 | Boys  n=3820 | Girls  n=3943 | p-value* |
| --- | --- | --- | --- | --- |
| Age, n (%) |  |  |  |  |
| 10 years | 3117 (40.2) | 1469 (38.5) | 1648 (41.8) | 0,005 |
| 11 years | 2540 (32.7) | 1263 (33.1) | 1277 (32.4) |  |
| 12 years | 2106 (27.1) | 1088 (28.4) | 1018 (25.8) |  |
| Place of living, n (%) |  |  |  |  |
| Village | 1651 (21.3) | 810 (21.2) | 841 (21.3) | 0,030 |
| City < 20k | 1393 (17.9) | 634 (16.7) | 759 (19.3) |  |
| City 20-100 k | 1530 (19.7) | 760 (19.9) | 770 (19.5) |  |
| City 100-500 k | 1825 (23.5) | 931 (24.3) | 894 (22.7) |  |
| City > 500 k | 1364 (17.6) | 685 (17.9) | 679 (17.2) |  |
| Screen time, n (%) |  |  |  |  |
| <2 h/day | 2708 (34.9) | 1217 (31.8) | 1491 (37.8) | <0.001 |
| 2-4 h/day | 2852 (36.7) | 1420 (37.2) | 1432 (36.3) |  |
| ≥4 h/day | 2203 (28.4) | 1183 (31.0) | 1020 (25.9) |  |
| Sleep time, n (%) |  |  |  |  |
| <6 h/day | 756 (9.7) | 355 (9.3) | 401 (10.2) | <0.001 |
| 6-8 h/day | 3885 (50.0) | 1834 (48.0) | 2051 (52.0) |  |
| ≥8 h/day | 3122 (40.2) | 1631 (42.7) | 1491 (37.8) |  |
| Physical activity level, n (%) |  |  |  |  |
| Low | 895 (11.5) | 491 (12.9) | 404 (10.2) | <0.001 |
| Moderate | 3376 (43.5) | 1436 (37.5) | 1940 (49.2) |  |
| High | 3492 (45.0) | 1893 (49.6) | 1599 (40.6) |  |
| Anthropometric measurements, x±SD |  |  |  |  |
| Body weight, kg | 42.6 ± 11.8 | 43.2 ± 12.4 | 41.9 ± 11.1 | <0.001 |
| Waist circumference, cm | 66.0 ± 10.4 | 68.2 ± 11.1 | 63.9 ± 9.2 | <0.001 |
| Height, cm | 149.9 ± 8.9 | 149.9 ± 8.9 | 149.8 ± 8.9 | 0.914 |
| BMI, Z-score | 0.008 ± 1.0 | 0.005 ± 1.1 | 0.011 ± 1.0 | 0.805 |
| BMI category, n (%) |  |  |  |  |
| Underweight (thinness) | 888 (11.4) | 433 (11.3) | 455 (11.5) | 0,955 |
| Normal weight | 5687 (73.3) | 2800 (73.3) | 2887 (73.2) |  |
| Overweight or obesity | 1188 (15.3) | 587 (15.4) | 601 (15.3) |  |
| WHtR (ratio), x±SD | 0.44 ± 0.06 | 0.45 ± 0.07 | 0.43 ± 0.06 | <0.001 |
| WHtR category, n (%) |  |  |  |  |
| WHtR<0.5 | 6459 | 2934 (76.8) | 3525 (89.4) | <0.001 |
| WHtR>=0.5 | 1304 | 886 (23.2) | 418 (10.6) |  |

* P-values were calculated using the Mann-Whitney’s test for continuous variables and the Chi-squared test for categorical variables.

**Additional File 2. Dietary outcomes of the study population (n=7763).**

| Variables | Total  n=7763 | Boys  n=3820 | Girls  n=3943 | p-value* |
| --- | --- | --- | --- | --- |
| pHDI (score), median (IQR) | 18.8 (13.5-26.7) | 18.3 (13.0-26.5) | 19.0 (13.7-26.7) | 0.003 |
| nHDI (score), median (IQR) | 9.2 (5.6-16.2) | 10.6 (5.6-16.4) | 9.0 (5.6-15.6) | 0.001 |
| DQI (score), median (IQR) | 8.7 (1.0-17.3) | 8.0 (0.2-16.3) | 9.6 (1.5-18.0) | <0.001 |
| Eating breakfast, n(%) |  |  |  |  |
| <1 days/week | 666 (8.6) | 294 (7.7) | 372 (9.5) | <0.001 |
| 1-3 days/week | 1170 (15.1) | 514 (13.4) | 656 (16.6) |  |
| 4-6 days/week | 1064 (13.7) | 511 (13.4) | 553 (14.0) |  |
| every day | 4863 (62.6) | 2501 (65.5) | 2362 (59.9) |  |
| Eating meals at school, n (%) |  |  |  |  |
| <1 days/week | 571 (7.4) | 329 (8.6) | 242 (6.1) | <0.001 |
| 1-3 days/week | 773 (10.0) | 398 (10.4) | 375 (9.5) |  |
| 4-6 days/week | 1365 (17.5) | 677 (17.7) | 688 (17.5) |  |
| every day | 5054 (65.1) | 2416 (63.3) | 2638 (66.9) |  |
| Eating meals with family, n (%) |  |  |  |  |
| not at all | 253 (3.3) | 124 (3.3) | 129 (3.3) | 0.334 |
| <1 days/week | 446 (5.7) | 205 (5.3) | 241 (6.1) |  |
| 1-2 days/week | 1151 (14.8) | 541 (14.2) | 610 (15.4) |  |
| 3-4 days/week | 1637 (21.1) | 809 (21.2) | 828 (21) |  |
| 5-6 days/week | 1254 (16.2) | 636 (16.6) | 618 (15.7) |  |
| every day | 3022 (38.9) | 1505 (39.4) | 1517 (38.5) |  |
| Nutritional knowledge, n (%) |  |  |  |  |
| Low | 2068 (26.6) | 1087 (28.4) | 981 (24.9) | 0.001 |
| Moderate | 4676 (60.3) | 2257 (59.1) | 2419 (61.3) |  |
| High | 1019 (13.1) | 476 (12.5) | 543 (13.8) |  |

* P-values were calculated using the Mann-Whitney’s test for continuous variables and the Chi-squared test for categorical variables.
